# Improving Coordination to Strengthen Operationalization of Malaria Surveillance and Routine Data Quality: Landscape Analysis of Current Surveillance-Related Initiatives

**DOI:** 10.4269/ajtmh.23-0460

**Published:** 2024-07-30

**Authors:** Selgün Kayaalpli, Hannah Margaret Edwards, Ann-Sophie Stratil, Julianna Smith, Médoune Ndiop, Balthazar Candrinho, Arantxa Roca-Feltrer

**Affiliations:** ^1^ Malaria Consortium, London, United Kingdom;; ^2^ Programme National de Lutte Contre le Paludisme, Dakar, Senegal;; ^3^ Programa Nacional de Controlo da Malaria, Maputo, Mozambique;; ^4^ PATH, Maputo, Mozambique

## Abstract

Improving the visibility and global coordination of malaria surveillance and data quality improvement initiatives is required to optimize sharing of best practices, tools, and approaches and to promote efficient, effective, and equitable distribution of resources. With these aims in mind, Rollback Malaria’s Surveillance, Monitoring and Evaluation Working Group established the Surveillance Practice and Data Quality Committee in May 2021. As a priority initiative, the committee conducted a landscape analysis of implementing partners’ (IPs’) malaria surveillance–related projects. A questionnaire that included questions on current project objectives, activities, geographic scope, and lessons learned was distributed among committee members and other IPs. Three years since its inception, information has been submitted regarding 49 projects by 25 IPs and funded by 17 donors. To present and share the landscaping results, an interactive dashboard was published to the Rollback Malaria’s Global Malaria Dashboard website (endmalaria.org) in March 2021. It is the first time that multiple stakeholders have shared such information regarding surveillance projects.

## INTRODUCTION

Surveillance is key in the fight against malaria as it helps health authorities make informed decisions on where to focus their interventions and how to mobilize their resources. Significant efforts have been made in developing normative guidance for surveillance since the WHO incorporated it as a core intervention in their Global Technical Strategy for Malaria 2016–2030^1^ and recently published a surveillance assessment toolkit that national malaria control programs (NMCPs) can use to assess their surveillance systems and track progress toward surveillance system strengthening.^2^ Effective surveillance systems require high-quality data collection, reporting, and analysis as well as strong governance and the ability to make timely and responsive decisions. There are, however, numerous challenges in operationalizing surveillance practices across malaria-endemic countries, including gaps in global coordination and alignment between stakeholders and implementing partners (IPs), resulting in fragmented resources and inadequate documentation of current funded initiatives.^3^ In response, Rollback Malaria’s (RBM’s) Surveillance, Monitoring and Evaluation Working Group (SME WG) established the Surveillance Practice and Data Quality (SP&DQ) Committee in May 2021 following recommendations from the 31st SME Working Group Annual General Meeting.^3^

**Figure 1.**
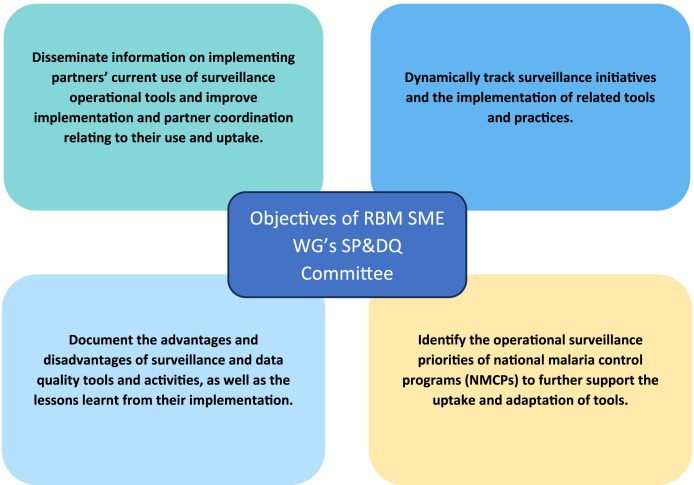
The four key objectives of RBM SME WG’s SP&DQ Committee. RBM = Rollback Malaria; SME WG = Surveillance, Monitoring and Evaluation Working Group; SP&DQ = Surveillance Practice and Data Quality.

The SP&DQ Committee of the RBM’s SME WG is composed of surveillance experts from NMCPs, IPs, and donor organizations with experience and interest in capacity building for surveillance, monitoring, and evaluation; data quality; data visualization; surveillance assessments; and surveillance tools, as well as other related fields. The SP&DQ Committee was established with four key objectives and the overall aim of improving the visibility, documentation, coordination, and harmonization of surveillance and data quality improvement initiatives, thereby increasing global awareness of malaria surveillance (Figure 1). Despite the particularities of the systems and the monitoring needs in each country, global coordination and alignment of support are important for sharing best practices and tools, facilitating cross-learning to overcome common challenges, and ensuring coordination in the distribution of resources.

The RBM’s Global Malaria Dashboards provide up-to-date information on some key elements of malaria control and elimination efforts in countries around the world to support informed decision-making, resource allocation, and advocacy.^4^ The first priority activity of the SP&DQ Committee was to create a new dashboard for this RBM initiative, called the Implementing Partners Surveillance Projects (IPSP) Dashboard, to provide information on the contributions of malaria surveillance efforts of key partner countries.^5^ These activities from IPs are designed to support NMCPs by providing additional technical and financial resources. Consolidating these efforts and maintaining an up-to-date repository in a single IPSP Dashboard, containing data on which partners and in which geographies partners are supporting surveillance-related projects, facilitate sharing best practices and lessons learned across a diverse range of stakeholders and helps improve transparency and alignment of investment efforts. The IPSP Dashboard is a tool that can be used by stakeholders to track surveillance projects and identify opportunities for collaboration, potential overlaps, and learning. The aim is that this information will complement additional work in progress from the SP&DQ Committee (planned in early 2024) to gather information about NMCPs’ key surveillance challenges and facilitate implementation of the WHO’s Surveillance Assessment Toolkit. Identified NMCP needs can then ideally be linked with appropriate IPs mapped out through the IPSP for funding and technical support needs.

## MATERIALS AND METHODS

Using the RBM’s SME WG platform for dissemination, a structured questionnaire was distributed among committee members and IPs to gather data on currently implemented projects, their objectives, specific activities, implementation areas, and lessons learned. The questionnaire required respondents to assign their activities to four thematic areas: “case surveillance,” “data to action,” “data quality,” and “governance” (Table 1).

**Table 1:** Activities included within the four thematic areas of surveillance projects

Thematic Area	Activity Type
Case Surveillance	Capacity Building Case Detection Reporting Frequency Resolutions of Reported Data Reporting and Data Management Tool Development
Data Quality	Capacity Building Tool Development Training
Data to Action	Capacity Building Data Analysis and Modeling Data Visualization Malaria Repository (system) Outbreak and Warning System Planning and Operations (data use) Stock Outs of Commodities Tool Development Stratification and Targeting
Governance	Capacity Building Coordination Human Resources Structure Operational Meetings Policy Quality Assurance Tool Development Training and Supervision

Activity types are specified within the Implementing Partners Surveillance Projects questionnaire.

The data collection questionnaire remains open for partner input at any time, but a refresher drive is planned on an annual basis. The initial drive for data collection was conducted over a 6-week period in September/October 2021 and resulted in a total of 10 organizations submitting information on 23 projects. After this initial data collection, the IPSP Dashboard was formed and published on the RBM’s Global Malaria Dashboards site in March 2022. The dashboard allows visualization of the global spread of projects by different partners, and information can be filtered by the user to look at specific countries, IPs, themes, and expected project end dates.

**Figure 2.**
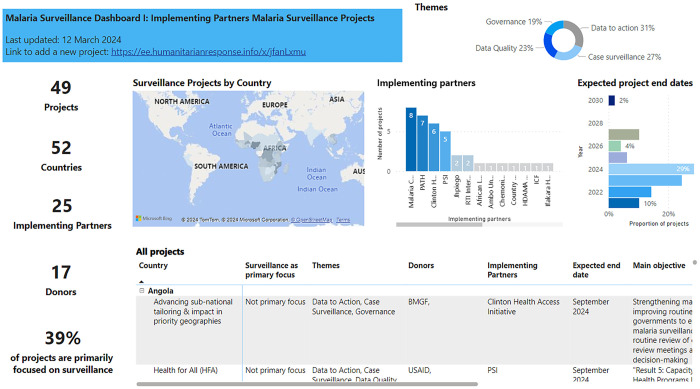
The IPSP Dashboard home page. The visualization can be filtered by country, implementing partner, thematic area, and project end date. IPSP = Implementing Partners Surveillance Projects.

The third-year data collection period was completed in December 2023. The number of submitted projects reached 49 by 25 IPs and was funded by 17 donors across 52 countries (Figure 2). Nigeria and Mozambique were the top two countries in terms of number of active projects, with IPs implementing 12 and 10 projects in each country, respectively. Burkina Faso and the Democratic Republic of Congo followed them, each with nine active projects. To our knowledge, this was the first time an effort was made to comprehensively capture the current landscape of surveillance-related projects and activities.

To strengthen the uptake of the IPSP questionnaire and dashboard, projects are showcased through the committee’s regular newsletter (circulated to around 800 contacts, including >50 NMCP contacts from 52 countries) with a dedicated section for IPs to present the details, successes, and lessons learned from the projects submitted. Identified use cases of the dashboard include the tracking of information to:

1. To target resources for the greatest impact by identifying areas where potential gaps in surveillance efforts exist: The IPSP Dashboard can help identify gaps or needs in malaria surveillance support, especially in “high-burden to high-impact” countries as defined by the WHO.^6^ By coordinating investments in strengthening surveillance systems in these areas, donors and IPs can assist countries in improving the accuracy and completeness of data, which can be used to better target interventions to prevent and control malaria.
2. To maintain an up-to-date repository of surveillance investments and to track the progress of projects over time: The dashboard contains details of current surveillance investments with detailed information on timelines, focus areas, geographic scope, and anticipated outcomes. This can help demonstrate the value of investments and encourage continued support for malaria control and elimination efforts.
3. To support advocacy efforts for increased investment: The data and information provided by the IPSP Dashboard, especially when they are tied to NMCPs’ stated needs, can be used to support advocacy efforts for increased investment in surveillance systems and collected data usage. By highlighting the progress of current investments and demonstrating the need for additional resources, stakeholders can advocate for increased attention and funding, as well as highlight investment gaps for certain countries.

As well as motivating annual updates to the dashboard, the committee’s next priorities include an analysis of stakeholder engagement with the dashboard to determine its uptake and utility to different partners. It is hoped that these findings will further improve and publicize the dashboard for greatest impact as well as to complement other RBM dashboards for a more comprehensive picture of malaria-related efforts. Furthermore, it is envisaged that integration of this partner information with needs assessments of the NMCPs through operationalization of the WHO’s Surveillance Assessment Toolkit will develop a pathway for targeting resources and efforts to address gaps in country surveillance systems.^2^

## CONCLUSION

In summary, this initiative represents the first time that donors, IPs, NMCPs and academic institutions have publicly shared “who is investing and supporting what and where”–type information in the frame of surveillance projects. It is not yet a comprehensive repository, as there is reporting bias influenced by the international partners already active within the SME WG platform through which participation has been encouraged. However, efforts are being made to reach out to more local partners involved in such efforts. The result of these efforts can be seen in the increase in projects submitted with each annual promotion of the dashboard. Further increasing this reach is a key priority over the coming rounds of data collection and efforts that have already been made to connect with civil society organizations. Information provided by the IPSP Dashboard and other components of the RBM’s Global Malaria Dashboards can be leveraged to ensure that investments in malaria control and elimination are targeted to where they are most needed and therefore have the greatest impact on reducing the burden of malaria.
